# Factors Affecting Disability Disclosure in Employment Setting for Individuals with Intellectual Disability

**DOI:** 10.3390/ijerph20043054

**Published:** 2023-02-09

**Authors:** Young-An Ra

**Affiliations:** School of Counseling Psychology and Social Welfare, Handong Global University, Pohang-si 37554, Republic of Korea; yar4466@handong.edu

**Keywords:** intellectual disability, disability disclosure, consensual qualitative research, vocational education

## Abstract

This study aimed to increase the understanding of this disability disclosure of people with intellectual disability by examining the determinants of their disclosure at work. For this aim, six individuals with intellectual disability were interviewed, and consensual qualitative research (CQR) was used to identify factors related to their disability disclosure. As results, the factors that affect the disability disclosure were largely divided into personal variables and environmental variables, and various factors including confidence, disability severity, employment type, employers, co-workers, and organizational culture were mentioned. The results of this study can help people have better understanding about disability disclosure in employment settings. We also discuss how vocational education for individuals with intellectual disability should be offered.

## 1. Introduction

For people with disabilities, finding employment and maintaining a job are an important transition in their lives [1], because having a job can be a decisive event for individuals with disabilities to keep a stable life. However, the high unemployment rate of people with disabilities has always been an obstacle. According to many studies, it has been reported that their income levels and life satisfaction are relatively lower than those of individuals without disabilities [2]. People with disabilities also noted that it is not easy for them to get and adjust to their jobs. Therefore, it is a very important and meaningful issue to increase the employment rate for people with disabilities and help them maintain their jobs for a prosperous life. In particular, employment for individuals with intellectual disabilities and their quality of life have received significant attention [3]. This is because people with intellectual disabilities are a group of people who struggle to maintain employment due to the perceptions that they have relatively low cognitive abilities and physical health compared to individuals without disability. Especially, for individuals with intellectual disabilities, there has been negative gaze and discrimination from others which are unavoidable problems in the employment process or in their workplace [4]. Therefore, there is a tendency to be reluctant to open up or explain their disability status to others and also to find employment while hiding their disability. Hiding disability status has been possible, because among the several types of disabilities, intellectual disability is invisible. Thus, for people with intellectual disability, they are able to find a job while concealing a mild intellectual disability status. However, there is also the disadvantage that if they hide their disability and get a job, they cannot receive proper job accommodations that they need due to their difficulties. Therefore, whether to hide the disability and get a job or to disclose and overcome social prejudice has always been a problem for many people with intellectual disabilities and their families.

Disability disclosure is defined as an act of intentionally disclosing one’s disability when applying for employment or in the workplace [5]. People with disabilities often face situations in which they need to open up about their disabilities in various employment environments, such as when applying for a job, interviewing, or starting a job. Many studies [5,6] report that employers have negative prejudices against applicants and employees with disabilities, which can put them at a disadvantage in the recruitment process and in their careers compared to other applicants and co-workers. As such, disability disclosure can have negative consequences due to prejudice or discrimination at the time of hiring, promotion, and job evaluation [7]. According to Baldrige and Veiga [8], the decision to disclose a disability in the workplace can damage one’s position or reputation after disclosure. Therefore, most previous studies reveal that the majority of people with intellectual disabilities do not disclose their disabilities when they are employed or in their careers due to fear of such prejudice [9].

Nonetheless, there are some cases in which some people with intellectual disabilities disclose their disability status during the employment process or at work [9]. In their study, Allen and Carlson [7] explained the reasons why people with intellectual disabilities disclose their disability to employers despite disadvantages such as discrimination or prejudice; they mentioned that job accommodations and work adjustments were the most important factors that lead to disability disclosure. According to Bassett et al. [10], explaining poor work productivity was another reason for disability disclosure. Other causes include to obtain help from others, to account for performance in the workplace, to account for a career break, and when disclosure is mandatory [10].

In conclusion, the dilemma between the negative prejudice related to disability disclosure and the benefits that can be obtained from it has been discussed in previous studies. Despite the importance of disability disclosure, there have been few studies on this topic and little research on which factors affect disability disclosure, especially for individuals with intellectual disability. Therefore, this study aimed to find out the factors that lead to disclosure of disability during employment or in the workplace. The research question addressed in this study is as follows.

What factors lead to disability disclosure in the employment environment for people with intellectual disabilities?

## 2. Methodology

### 2.1. Participants

In this study, participants were asked to participate in the research after explaining the purpose of the study to affirm the confidentiality of their information. Participants were people with intellectual disabilities who had experience of employment. A total of 6 participants responded to the interview request, 5 men and 1 woman. All of them were recruited from a social welfare organization in Korea. Their age rage was from 20 to 31. Their degrees of disability were considered as mild, which means that their intellectual quotient ranged from 50 to 70. More information is indicated in Table 1.

### 2.2. Data Collection

Interview questions were developed based on previous research regarding disability disclosure [5,7,8,9,10]. They were developed in a semi-structured format, and the content validity was verified by researchers who had rehabilitation counseling PhDs. An interview of about an hour was conducted in person for each participant. Once participants were ready, interviews were conducted in a class at a social welfare organization. The interview guide included contents such as leading the interview inductively rather than deductively, developing an interview protocol with open-ended questions, and gathering works, narratives and studies rather than numbers [11]. Examples of an interview question were, “Do you think disability disclosure is necessary for employment?” and “What are the factors that lead disability disclosure in your employment setting?”

### 2.3. Data Analysis

After transcribing the interview contents from participants, the consensual qualitative research (CQR) method of Hill, Thomson, and Williams [11] was used for data analysis in this study. CQR is a qualitative research method which provides rich and in-depth descriptions of individuals’ experiences. For data analysis, three researchers majoring in counseling psychology participated. Data analysis in this study was conducted through the following process: (1) developing domains to reflect the interview contents, (2) constructing core ideas, which involves editing the participants’ words into clear cases, (3) cross-analysis, which is the process of agreement among research team members, and (4) stability check, the final stage of checking the results. These processes were conducted according to a careful procedure of consensual qualitative research (CQR) suggested by Hill et al. [11].

## 3. Results

As results, research participants revealed that there are two domains, personal and environmental, as determinants of their disability disclosures. Table 2 summarizes the central concepts and contents given by the research participants for these areas.

### 3.1. Domain 1: Personal

Within the personal domain, there were three core ideas, confidence, disability severity, and employment type. Confidence and disability severity were considered to be typical and employment type was considered to be variant. Each one is discussed below.

Confidence. The core idea of confidence was mentioned as an important factor in predicting the discrimination or disadvantages that individuals with intellectual disabilities would face when they disclose their disability. Participants reported that being able to predict unfair treatment related to disability disclosure in advance and confidence in how well they can handle the disadvantages lead to disability disclosure.

“Confidence is one of the most important. The confidence to disclose the disability and to overcome the prejudice...”

Participant 6

Disability Severity. The research participants mentioned that disability severity is also important as a determinant of disability disclosure. Work productivity was also mentioned in relation to this disability severity; even if an individual with a disability does not want to disclose his or her disability status, there is a huge difference in work productivity depending on the disability severity they have. They reported that disability status needs to be disclosed to the company or an employer in order to explain their speed and efficiency of the work.

“I have to tell them about my disability because I’m slow at work. I need to explain and ask for understanding.”

Participant 1

Employment Type. The participants also reported that employment type is also an important factor for disability disclosure. They mentioned that whether they are full-time or part-time at work has an impact on their ability to disclose their disability. Participants reported that as part-time workers they felt less obligated to disclose their disability, and full-time workers were more willing to disclose their disability.

“If it’s a full-time job, then my disability should be disclosed. They will find out anyway when I work for a long time.”

Participant 4

### 3.2. Domain 2: Environmental

Within the environmental domain, there were also three core ideas, employers, co-workers, and organizational culture. Employers and co-workers were considered to be typical and organizational culture was considered to be variant. Each one is discussed below.

Employers. Participants mentioned in their interviews that the employer’s attitude and support were important factors for their disability disclosure. They reported that how employers think about their disability has a significant effect on disability disclosure; it has been difficult to disclose their disability if the employer had a negative perspective about disability and hiring individuals with intellectual disability.

“It’s harder to say if the employer has a bias about disability. If I can think that the employer is someone who can understand my situation, it will be easier for me to disclose my disability.”

Participant 2

Co-workers. Participants noted that their co-workers’ attitude toward intellectual disability is also a significant factor that influenced their decision on disability disclosure. They mentioned that how co-workers at work think about disability and whether they have negative thoughts about working with people with intellectual disabilities in the same work environment have an impact on their disability disclosures.

“It is also important what my colleagues think of me. They are the people I work with and spend a whole day together at workplace.”

Participant 5

Organizational Culture. Organizational culture was also an important consideration when the participants consider their disability disclosure. They said that they can disclose their disability more easily to companies which support disability-related work or disability-friendly culture.

“There are organizations that are open about disability. If I work a lot with people with disabilities or work related to disabilities, I can disclose my disability more easily. That is depends on the organizational culture.”

Participant 6

## 4. Discussion

The purpose of the present study was to identify the factors that lead disability disclosure of individuals with intellectual disability at their work. According to the results of consensual qualitative research (CQR), the disability disclosure determinants were in two domains, personal variables and environmental variables. Personal variables were confidence, disability severity, and employment type. The environmental variables were employers, co-workers, and organizational culture.

Participants mentioned that confidence in whether they could handle the prejudice or disadvantages they might receive after disclosing their disabilities was an important factor of their disability disclosure. Existing studies [1,9,12] also found that the most important factor for people with disabilities to be reluctant to reveal their disabilities is the negative perspective of other people. The participants in this study were also most concerned about this stigma, discrimination, and unfair treatment from others when they disclose their disabilities. Thus, how confident they are that they will endure those discriminations plays a very important role in disclosing their disabilities.

In addition, participants with intellectual disabilities noted that that the severity of disability is also a factor influencing disability disclosure. They mentioned visible and invisible disabilities; intellectual disability is not a disability that anyone can recognize when looking at a person, so they think more seriously about disclosing their disability. In a previous study, it was said that the decision on whether or not to disclose disability could be a more important issue, especially for those with invisible disabilities [5]. Participants mentioned that if their intellectual disabilities are mild, so they can do their work well without any problems, they would not choose to disclose their disability status. However, if their disabilities are severe they may need to disclose their situations to achieve appropriate job accommodations or work adjustment advantages.

Employment type was also an important variable for disability disclosure. Regarding the employment type, the participants mentioned that whether they are full-time employees or part-time workers affects disability disclosure. They reported that they do not feel compelled to disclose their disability when they are employed in part-time work, because it was not a place to work for a long time. They mentioned that there was no need to disclose their disabilities unless absolutely necessary in part-time work. On the other hand, if an individual with intellectual disability is employed in a full-time job, he or she feels more obligated to disclose the disability.

For the environmental variables, three factors were employer, co-workers, and organizational culture. In this study, the participants reported that their employer is an important person for disability disclosure. They noted that it would be difficult to disclose their disabilities if they felt that their employers had prejudice about disability [1,3]. On the other hand, if their employer is a person who does not have any discrimination toward the disability, they would not have much trouble deciding on disability disclosure.

Co-workers were also a factor that had an important influence on disability disclosure for individuals with intellectual disabilities. Participants reported that their colleagues’ attitudes toward disability, as well as their employer’s, were a factor to consider in their disability disclosure decisions. They noted that they would be more likely to open up about their disability if their peers were unbiased and supportive of their disability, but would be reluctant to disclose their disability if co-workers viewed disability in a hostile or negative way.

The last factor of the environmental variables is organizational culture. Organizational culture is shared assumptions that guide what happens in organizations by explaining appropriate behavior for various situations [13]. Participants reported that the more they worked in a company with a disability-friendly culture, the easier it would be to disclose their intellectual disability. This is consistent with a previous study [14] that found that people with disabilities who work in places related to their disabilities can work with less discrimination than in other employment environments. Participants with intellectual disabilities recognized organizational culture as a factor influencing disability disclosure.

The results of this study would be able to assist us to think about the prejudice and discrimination that individuals with intellectual disability may receive due to their disability disclosure. Many employees with intellectual disabilities believe that their colleagues, employers, or others are prejudiced against them. The results of the present study obviously indicated that social prejudice and discrimination influenced the decision on disability disclosure for people with intellectual disability. Moreover, because South Korean culture is generally considered homogenous and conservative when it comes to customs and behavior, individuals with intellectual disability in Korea may not be able to take an active part in their work. Previous studies [15,16] also reported that the biggest factor hindering the disclosure of disability at work was negative gaze or discrimination, and direct or indirect stigma they received from others. This stigma causes severe psychological effects for individuals with intellectual disability; it led to anger, alienation, frustration, and in the long term, eventually sadness. Prince and Prince [17] also showed that the stigma and discrimination experienced by people with disabilities have a great impact on their self-esteem, and whether they can exist as members of society. Therefore, positive experiences related to disability disclosure are important. The experience of not being disadvantaged despite the disability disclosure will boost self-confidence among employees with intellectual disabilities. The results of this study will help them to have better understanding about their experience of disability disclosure at their work.

In addition, the present study will help researchers to find effective practices or measures for assisting individuals with intellectual disability and to provide better support services for them. In terms of how to change work environments and social systems for individuals with intellectual disability, providing regular vocational education to improve awareness of people with disability can be an important opportunity. This continuous education would be able to assist individuals with intellectual disability to work in a safe and pleasant work environment.

There are limitations of the present study. The study only recruited participants with intellectual disability who had experiences of disability disclosure, not including individuals who did not choose to disclose their disabilities. Therefore, the results may not be generalized easily. Researchers need to examine this topic in-depth, considering both sides, disclosed and undisclosed disability. Additionally, racial or gender differences in disability disclosure can be explored to understand the decision better.

## 5. Conclusions

The purpose of this study was to explore the phenomenon of disability disclosure of individuals with intellectual disability in employment and vocational rehabilitation settings. According to the results, it was found that confidence, disability severity, employment type, employer, co-workers, and organizational culture have a great influence on disability disclosure for them. People with intellectual disabilities and vocational education professionals who assist them need to have a deep understanding of these topics in order to be successful in disability disclosure decisions that may be faced in employment processes or the workplace. The results of this study suggest that disability disclosure should be decided based on a careful understanding of the consequences of it; if they choose to disclose their disabilities, individuals with intellectual disability themselves and educational professionals need to help them and also should highlight the positive aspects of the disability disclosure to prepare for the results of it. This would be able to help employees with intellectual disability adjust well in their workplace and lead to higher productivity at work.

## Figures and Tables

**Table 1:** Information about Interview Participants.

	Name (Alias)	Age	Gender	Degree of Disability	Work Experience (y)
1	Andy	20	M	Mild	1
2	Chris	25	M	Mild	2
3	David	29	M	Mild	4
4	Harry	30	M	Mild	5
5	James	31	M	Mild	5
6	Cindy	30	F	Mild	5

**Table 2:** Summary of Domains, Core Ideas, Contents, and Frequencies.

Domain	Core Idea	Content	Frequency
Personal	Confidence	Confidence in overcoming discrimination	Typical (5)
	Disability Severity	Work productivity due to the disability severity	Typical (5)
	Employment Type	Full-time or part-time job	Variant (2)
Environmental	Employer	Employer’s attitude toward the disability	Typical (6)
	Co-workers	Co-workers’ attitude toward the disability	Typical (5)
	Organizational Culture	Disability-friendly culture	Variant (2)

## Data Availability

The data that were used in the present study can be provided by the corresponding author upon reasonable request.
